# Longitudinal Monitoring Reveals Persistence of Colistin-Resistant *Escherichia coli* on a Pig Farm Following Cessation of Colistin Use

**DOI:** 10.3389/fvets.2022.845746

**Published:** 2022-03-14

**Authors:** Nwai Oo Khine, Kittitat Lugsomya, Waree Niyomtham, Tawat Pongpan, David J. Hampson, Nuvee Prapasarakul

**Affiliations:** ^1^Department of Veterinary Microbiology, Faculty of Veterinary Science, Chulalongkorn University, Bangkok, Thailand; ^2^The International Graduate Program of Veterinary Science and Technology (VST), Faculty of Veterinary Science, Chulalongkorn University, Bangkok, Thailand; ^3^Department of Infectious Diseases and Public Health, Jockey Club College of Veterinary Medicine and Life Sciences, City University of Hong Kong, Kowloon, China; ^4^Center of Excellence in Diagnosis and Monitoring of Animal Pathogens (DMAP), Bangkok, Thailand; ^5^School of Veterinary Medicine, Murdoch University, Perth, WA, Australia

**Keywords:** colistin resistance, *Escherichia coli*, *mcr* genes, longitudinal monitoring, pigs

## Abstract

Colistin-resistant bacteria harboring plasmid-mediated *mcr* genes are of concern as they may be a cause of serious nosocomial infections. It is hypothesized that cessation of colistin use as a feed additive for pigs will reduce the occurrence and distribution of *mcr* genes in farms. The aim of this study was to investigate this hypothesis by longitudinal monitoring and characterizing of *mcr* positive *Escherichia coli* (MCRPE) isolates after colistin was withdrawn on a central Thailand pig farm that previously had a high frequency of MCRPE. Colistin use ceased at the beginning of 2017, and subsequently 170 samples were collected from farrowing sows and suckling piglets (*n* = 70), wastewater (*n* = 50) and farm workers (*n* = 50) over a 3.5-year period. Bacteria were identified by MALDI-TOF mass spectrometry and minimal inhibitory concentrations were determined by broth microdilution. The antibiogram of *mcr* positive *E. coli* isolates was determined using the Vitek2 automated susceptibility machine, and multiplex and simplex PCRs were performed for *mcr*-1–8 genes. MCRPE containing either *mcr*-1 or *mcr*-3 were isolated from pigs throughout the investigation period, but with a declining trend, whereas MCRPE isolates were recovered from humans only in 2017. MCRPE were still being recovered from wastewater in 2020. Most MCRPE isolates possessed the virulence genes *Stap, Stb*, or *Stx2e*, reflecting pathogenic potential in pigs, and showed high rates of resistance to ampicillin, gentamicin and tetracycline. Pulsed-field gel electrophoresis and multi-locus sequence typing showed that diverse MCRPE clones were distributed on the farm. The study identified a decline of pathogenic MCRPE following withdrawal of colistin, with pigs being the primary source, followed by wastewater. However, short-term therapeutic usage of other antibiotics could enhance the re-occurrence of *mcr-*carrying bacteria. Factors including the environment, management, and gene adaptations that allow maintenance of colistin resistance require further investigation, and longer-term studies are needed.

## Introduction

Colistin (polymyxin E) is one of the World Health Organization's highest priority antimicrobials: it is regarded as a last resort antibiotic, and is the treatment of choice for multidrug-resistant *Enterobacteriaceae* infections (1). Unfortunately, the emergence of mobile colistin resistance genes of the *mcr* gene family has jeopardized the efficacy of colistin. The plasmid mediated colistin resistance gene (*mcr-*1) was firstly identified in *E. coli* of porcine origin from China (2). Subsequently, other *mcr* variants including *mcr-*2 to *mcr-*10 were discovered mainly from members of the *Enterobacteriaceae* family (3–6) from different geographical areas (7, 8). The *mcr* genes encode phosphoethanolamine transferases enzymes which change the lipid A portion of the lipopolysaccharides (LPS), suppressing colistin binding (9). The *mcr* genes have been reported not only from various livestock origins (pigs, poultry, bovine) (10, 11) and food products (12) but also from the environment as well as from humans (13). Since the extensive usage of colistin in livestock farms played a major role in the occurrence of colistin resistant *mcr* genes, controlling the dissemination of these resistant genes from farms to the environment has become a critical concern (14). Moreover, *mcr* genes could be co-located with other important antibiotic resistance genes such as Extended spectrum beta-lactamase (ESBL) and carbapenemase genes (15, 16). These reports raised awareness of colistin usage and the challenge to clinical medicine.

In the swine industry, colistin had been applied therapeutically and/or prophylactically in several countries (17). Because of the importance of colistin usage in clinical infections, many countries have restricted the prophylactic usage of colistin in pig productions (18). Following the first identification of *mcr*-1 during nation-wide surveillance in Thailand (19), from the start of 2017 the Department of Livestock and Development (DLD) has prohibited prophylactic use of colistin sulfate in pig farms. Data regarding colistin resistance in bacteria from livestock in Thailand is still limited, although *mcr* positive *E. coli* have been detected in pig slaughterhouses from the Thailand border areas even after implementation of the colistin withdraw policy (20). Therefore, currently it is still debatable how this withdrawal of colistin usage may have influenced the emergence and spread of *mcr* genes in pigs and in the farm environment (21). It is thought to be unlikely that resistance plasmids can be entirely eliminated from bacterial populations (22). Previous studies have found withdrawal it was likely to be beneficial in controlling the emergence of *mcr*-1 in pigs once the selective pressure is removed (21, 23). However, the variations in duration of *mcr* gene persistence after cessation of colistin usage and the rate of dissemination from pigs to the farm environment are still concerning. Moreover, resistant bacteria from livestock can potentially spread to farmers or the environment resulting in the occurrence of antibiotic resistance genes (ARG). Furthermore, some studies have shown that even after drastic reductions of antibiotic use on farms, antibiotic resistant bacteria could be maintained in the farm by various factors (24–26).

The aim of this study was to determine the occurrence and extent of persistence of MCRPE following the cessation of colistin sulfate use on a representative pig farm which had a history of a high prevalence of MCRPE (19). Representative *E. coli* isolates that contained *mcr* genes were obtained from pigs, wastewater and farm workers over the study period and were characterized for antimicrobial susceptibility patterns, virulence factors, plasmid replicons, and clonal relationships.

## Materials and Methods

### Study Area and Farm Selection

A typical industrial pig farm with more than 1,000 breeder sows located in the central area of Thailand was selected for use in this study. Prior to 2017, colistin sulfate had been administered routinely to all suckling piglets from birth to weaning to prevent and control diarrhea. It was given via the water at a dose of 10 mg/kg body weight. The farm withdrew prophylactic colistin use in piglets from the beginning of 2017, following the guidance of the DLD. The farm management systems were not otherwise altered, and they continued to follow the recommendations of the Thai standard livestock farm criteria. Piglets with diarrhea were separated from healthy piglets by placing them in separate pens until they recovered. In cases of diarrhea in breeding sows and piglets, antibiotic injections including gentamicin, ceftriaxone, and/or penicillin/streptomycin combinations were used for treatment of individual animals.

### Sample Collection and Processing

The number and types of samples (from pigs, wastewater and humans) that were obtained are summarized in Table 1. Sample sizes were calculated based on the prevalence of *mcr* genes detected in the pig farm from our previous study (19) by using Epitools program http://epitools.ausvet.com.au. Samples were collected at five-time points spanning a 3.5 year period from cessation of colistin use: June 2017, September 2018, March 2019, April 2019, and June 2020.

**Table 1:** Details of the sample types and numbers collected at five different sampling times between 2017 and 2020, and numbers of samples found positive for MCRPE.

**Year**	**Type of sample**	**Sampling time**	**Number**	**Age of pigs at time of sampling**	**Samples positive for MCRPE**
2017	Farrowing sows	1	10	1–3 years	9
	Suckling piglets	1	5	21 days	0
	Wastewater (Before-biogas treatment)	1	5	–	2
	Wastewater (After biogas treatment)	1	5	–	0
	Farm workers	1	10	–	4
2018	Farrowing sows	2	10	1–3 years	5
	Suckling piglets	2	0	–	0
	Wastewater (Before-biogas treatment)	2	5	–	1
	Wastewater (After biogas treatment)	2	5	–	0
	Farm workers	2	10	–	0
2019 March	Farrowing sows	3	10	1–3 years	0
	Suckling piglets	3	5	21 days	0
	Wastewater (Before-biogas treatment)	3	5	–	0
	Wastewater (After biogas treatment)	3	5	–	0
	Farm workers	3	10	–	0
2019 April	Farrowing sows	4	10	1–3 years	1
	Suckling piglets	4	5	21 days	0
	Wastewater (Before-biogas treatment)	4	5	–	0
	Wastewater (After biogas treatment)	4	5	–	0
	Farm workers	4	10	–	0
2020	Farrowing sows	5	10	1–3 years	0
	Suckling piglets	5	5	21 days	5
	Wastewater (Before-biogas treatment)	5	5	–	5
	Wastewater (After biogas treatment)	5	5	-	1
	Farm workers	5	10	-	0

Approximately 25 g of fecal samples were collected from parity 1–6 farrowing sows, aged between 1 to 3 years (*n* = 50). Rectal swab samples from 21-day old suckling piglets belonging to the sows that were sampled were also collected (*n* = 20). Each farrowing sow with their respective litters were kept in farrowing pens, and at each visit one or two sows were sampled from each zone of the farrowing house. The same pens were visited at each sampling time, although the same sows were not necessarily sampled because of animal movements. In September 2018, only fecal samples from sows were collected since at the time of sampling the newly weaned piglets had been moved to another farm.

Wastewater samples (*n* = 50) from the wastewater tanks on the farm were collected, with 10 samples obtained at each visit. The wastewater was composed of pig manure along with the water used to clean the pig housing. Approximately 500 ml volumes were collected from wastewater tanks located before and after-biogas treatment, which were sited close to the sampled pig pens. The biogas process involves anaerobic fermentation by fermentative, acetogenic, and methanogenic bacteria to produce methane, carbon dioxide, hydrogen, and hydrogen sulfide gases. In addition, at the request of the company, at each sample collection time the farm submitted rectal swab samples from the same 10 farm workers for routine diagnostic purposes (*n* = 50).

Sampling from the pigs and the wastewater was conducted by an authorized veterinarian for the farm. The biohazard execution control was approved by the Institutional Biosafety Committee of the Faculty of Veterinary Science, Chulalongkorn University (IBC 2031011). The wastewater sample collection protocol was applied according to HACH water analysis guidelines (27).

### Bacterial Isolation and Identification

All samples were enriched in EC broth (Difco) containing 2 μg/ml colistin sulfate at a 1:9 ratio and incubated at 37°C overnight. The sample suspensions were grown on eosin-methylene blue (EMB) (Oxoid) agar containing 2 μg/ml colistin sulfate and were incubated overnight. One to three representative colonies with a characteristic metallic sheen on the EMB plates were randomly chosen and sub-cultured on tryptic soy agar (TSA) (Difco) from the samples from which growth was obtained. The colonies were identified as *E. coli* using IMViC biochemical tests and Matrix-Assisted Laser Desorption Ionization combined with time of-flight analysis (MALDI Biotyper, Bruker, USA), according to the manufacturer's recommendations (28). For minimal inhibitory concentration (MIC) determinations, antibiotic susceptibility testing, and PCR detection for virulence genes and plasmid replicon types, a single representative isolate from each positive sample was used.

### Antimicrobial Susceptibility Testing

The MIC for colistin was determined using the broth microdilution technique following CLSI guidelines (29). An MIC value of >2 μg/ml was considered to indicate colistin resistance (29). The antibiogram for *E. coli* isolates was determined using the AST-GN 38 test kit in a Vitek2 compact automated susceptibility level detection apparatus (BioMérieux, France). The antimicrobial groups that were included in Vitek2 were synchronized with veterinary guidelines (30). The 18 antimicrobials comprised amikacin (AK), amoxicillin (AMX), amoxicillin/clavulanic acid (AMC), ampicillin (AMP), cefalexin (CEX), cefpodoxime (CPD), cefovecin (INN), ceftiofur (XNL), chloramphenicol (C), enrofloxacin (ENR), gentamicin (GEN), imipenem (IMP), marbofloxacin (MBR), nitrofurantoin (NIT), piperacillin (PIP), tetracycline (TET), tobramycin (TOB), and trimethoprim/sulfamethoxazole (SXT). The MIC interpretations from the Vitek2 machine system (version-9) were made according to the Food and Drug Administration recommendations (31), CLSI guidelines (32) and EUCAST values (33). *E. coli* ATCC 25922, *Pseudomonas aeruginosa* ATCC 27853, and *Staphylococcus aureus* ATCC 25913 were used as the control strains.

### Detection of Plasmid-Mediated Colistin Resistance Genes

Genomic DNA was extracted from all available MCRPE isolates using the Thermo Scientific GeneJET Genomic DNA Purification Kit (Thermo Fisher Scientific). Multiplex-PCR was used to detect *mcr*1-5 genes, following a previously published protocol (34). *E. coli* strain CUP13 (35) that is positive for *mcr-*1 and *mcr-*3 as confirmed by Sanger sequencing was used for the positive control, and *E. coli* ATCC25922 was the negative control. The PCR conditions for *mcr* 6, 7, and 8 were adjusted and performed according to a previous description (4).

### Plasmid Replicon Typing

The 18 plasmid replicon types of *Enterobacteriaceae* were investigated by a set of multiplex and simplex PCRs. The primers used and the PCR conditions followed previously described methods (36). Briefly, PCR amplification, except the F-simplex, were conducted at 94°C for 5 min, followed by 30 cycles at 94°C for 1 min, 60°C for 30 s, 72°C for 1 min. The amplification was concluded with an extension program of 1 cycle at 72°C for 5 min. The PCR for F-simplex was performed in the same way except for annealing at 52°C.

### Detection of Virulence Genes

The *mcr* positive *E. coli* were examined for virulence genes that are commonly present in enterotoxigenic *E. coli* (ETEC) and enterohaemorrhagic *E. coli* (EHEC) by using previously described PCRs (37). Previously sequenced ETEC and EHEC strain were used as positive controls (38). The PCR assays were performed with GoTaq^®^ green master mix (Promega, USA) with the thermocycler conditions being an initial denaturation at 94°C for 10 min, followed by 30 cycles of denaturation at 94°C for 30 s, and annealing at 55°C for 45 s. Extension was at 72°C for 1.5 min increased by 3 s each cycle, followed by a final extension at 72°C for 10 min.

### Conjugation Assay

To determine whether *mcr* genes were located on transmissible plasmids, and their transferability rate, conjugation assays were performed by the broth mating technique (39). All the *mcr* positive strains detected by PCR were designated as donors, and *E. coli* J53, resistant to sodium azide, was used as the recipient strain. This recipient *E. coli* J53 strain is negative for fertility factors, is resistant to sodium azide (MIC >512 μg/ml) and is sensitive to colistin (MIC <2 μg/ml). Briefly, an overnight culture of bacterial colonies was diluted in Lysogeny broth (LB) and adjusted to OD600 value 1. A 1:1 ratio of donor and recipient then was mixed to obtain a final volume of 2 ml which was incubated overnight. Ten-fold serial dilutions of the overnight mixture were plated on LB agar (Oxoid) plates containing colistin (2 μg/ml) and sodium azide (100 μg/ml). The plates were incubated at 37°C for 2 days, and the transconjugant colonies were counted. The rate of conjugal transfer frequencies was calculated by dividing the number of transconjugant colonies by the number of donor colonies (40). Phenotypic colistin susceptibility testing, PCR detection of *mcr* genes and replicon type detections were repeated on the transconjugants.

### Pulsed-Field Gel Electrophoresis (PFGE)

To investigate clonal relatedness, PFGE was performed on all 65 available MCRPE isolates from the 33 positive samples (one to three isolates per sample), following the Centers for Disease Control and Prevention standard protocol (41). Briefly, overnight cultures of *E. coli* isolates were suspended in cell suspension buffer, and the cells were treated with proteinase K and mixed with the agarose gel solution. The gel plugs then were treated with lysis solution, and DNA in the plugs was digested with restriction enzyme *Xba*I (Thermo Scientific). Gel electrophoresis was undertaken using a Bio-Rad CHEF-DRIII system, with a 200 V field at an angle of 120° run for 17–20 h, incorporating *Salmonella* serovar Braenderup H9812 DNA as a standard. Dendrograms were created using the GeneTool program (Syngene, India) and analyzed by the GeneDirectory program (Syngene, India).

### Multi-Locus Sequence Typing (MLST)

A representative isolate from each of the 34 PFGE pulsotypes that were identified was randomly selected and included for MLST typing. A simplex PCR was performed for each of the 7 housekeeping genes of *E. coli* used in the Achtman MLST scheme (42). These genes encoded isocitrate/isopropyl malate dehydrogenase (*icd*), ATP/GTP binding motif (*recA*), adenylate kinase (*adk*), DNA gyrase (*gyrB*), malate dehydrogenase (*mdh*), adenyl succinate dehydrogenase (*purA*) and fumarate hydratase (*fumc*). The sequences were obtained using the Sanger sequencing platform. The *E. coli* MLST database at http://mlst.warwick.ac.uk/mlst/dbs/Ecoli was used to determine allele and sequence types (STs).

### Data Analysis

The colistin-resistance rates and virulence gene profiles for the representative isolates were described as percentages compared to different sources in each sample collection. The *mcr* positive rates among the samples and the association between each sample collection time were analyzed using Fischer's Exact Test (*p* ≤ 0.05).

## Results

### Detection of Colistin Resistant *E. coli*

**Figure 1:**
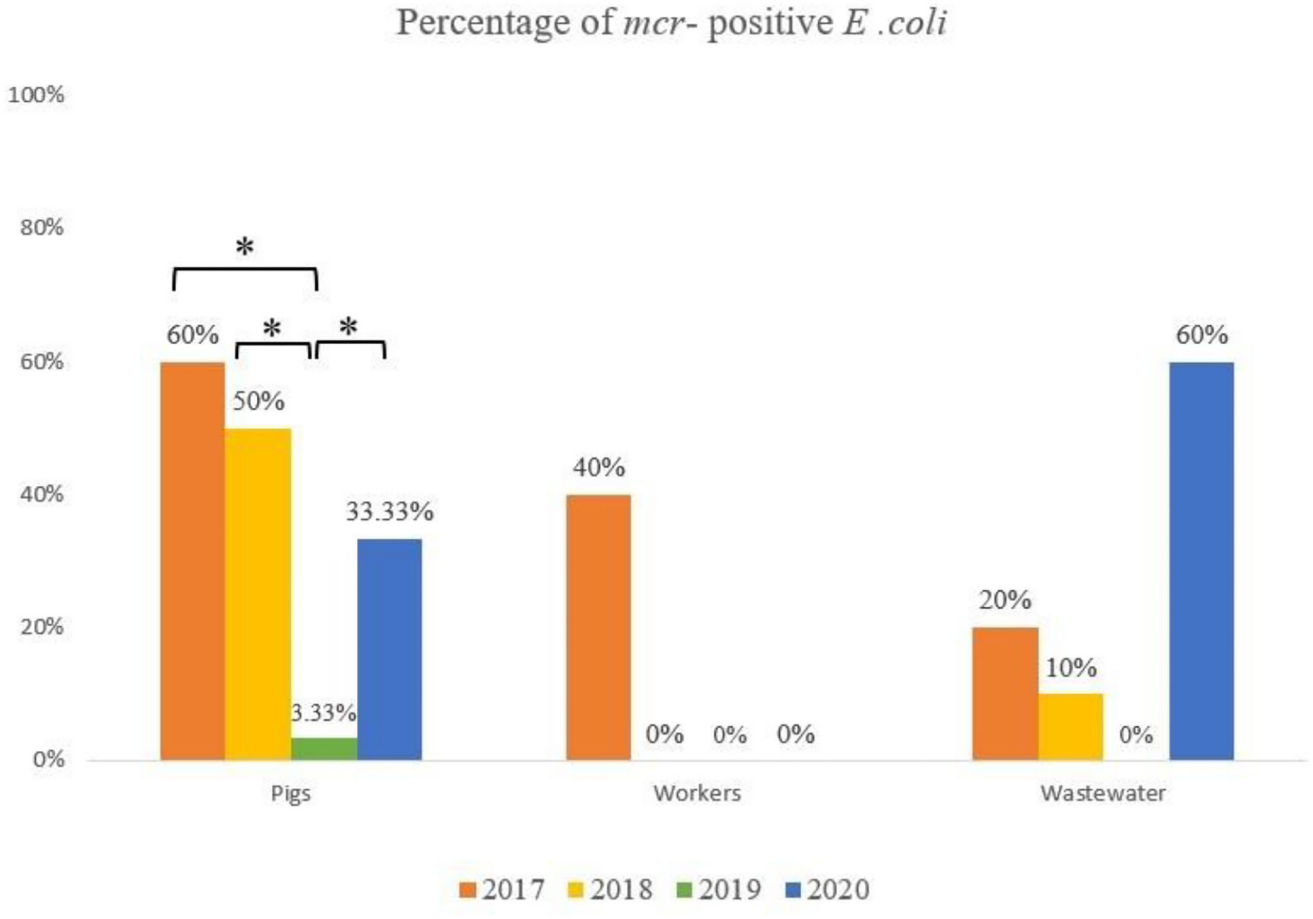
Comparison of the rate of *mcr* positive *E. coli* isolated from pigs, workers and the environment in four sample collection years (*significant difference; *p* ≤ 0.05).

A total of 33 of the 170 samples (20.6%) yielded colistin-resistant *E. coli*, and their MICs to colistin varied from 4 to 8 μg/ml. These positive samples were isolated from pigs (*n* = 20/70, 28.6%), wastewater (*n* = 9/50, 18%) and humans (*n* = 4/50, 8%). A comparison of the prevalence of MCRPE isolates for each sample type over the 3.5 years since colistin cessation is shown in Figure 1, and detailed information about the isolates is presented in Table 2. In pigs the high prevalence found in 2017 (60%) and 2018 (50%) was followed by only a single isolate recovered in 2019 (3.3%), and then another increase in 2020 (33.3%). In humans, resistant isolates were only found in 2017 (40%), while a comparatively low rate of positivity in wastewater in 2017 (20%) and 2018 (10%) was followed by none in 2019, and a high prevalence in 2020 (60%). The majority (8/50: 16%) of MCRPE isolates recovered from wastewater were obtained from samples taken before biogas treatment, with only one isolate recovered in 2020 being from a sample taken after the biogas treatment plant (Table 1).

**Table 2:** Characterization of 33 colistin-resistant *mcr* positive *E. coli* isolates from different years and sources.

**Collection date**	**Source and number sampled**	**Number of resistant isolates obtained**	***mcr* genes in resistant isolates**	**Virulence genes in *mcr* positive isolates**
2017	Pigs (*n =* 15)	9/15 (60%)	*mcr*-1 (8/15, 53.3%) *mcr*-3 (1/15, 20%)	*StaP*-*Stb* (5/9, 55.6%) *StaP*-*Stb*-*Stx2e* (1/9, 11.1%) Non-pathogenic (3/9, 33.3%)
2017	Humans (*n =* 10)	4/10 (40%)	*mcr*-1 (4/10, 40%)	*StaP*-*Stb* (2/4, 50%) Non-pathogenic (2/4, 50%)
2017	Wastewater (*n =* 10)	2/10 (20%)	*mcr*-1 (2/10, 20%)	*Stb* (2/2, 100%) Non-pathogenic (0%)
2018	Pigs (*n =* 10)	5/10 (50%)	*mcr*-1 (3/10, 30%) *mcr*-3 (2/10, 20%)	*Stb* (2/5, 40%) Non-pathogenic (3/5, 60%)
2018	Wastewater (*n =* 10)	1/10 (10%)	*mcr*-1 (1/10, 10%)	*Stb* (1/1, 100%) Non-pathogenic (0%)
2019	Pigs (*n =* 30)	1/30 (3.33%)	*mcr*-1 (1/30, 3.33%)	Non-pathogenic (100%)
2020	Pigs (*n =* 15)	5/15 (33.3%)	*mcr-*1 (5/15, 33.3%)	*Stb* (4/5, 80%) Non-pathogenic (1/5, 20%)
2020	Wastewater (*n =* 10)	6/10 (60%)	*mcr-*1 (6/10, 60%)	*Stb* (2/6, 33.3%) Non-pathogenic (4/6, 66.7%)

### Identification of Plasmid-Mediated Colistin Resistance Genes

Of the colistin resistant isolates obtained in 2017, the *mcr*-1 gene was detected in eight of the pig isolates, while *mcr*-1 and *mcr-*3 were detected together in two of these, and *mcr*-3 alone in one pig isolate. At the same time, *mcr*-1 was detected in all four of the isolates from workers and in both the isolates from wastewater samples (Table 2). In 2018, after colistin withdrawal for one and a half years, *mcr*-1 was detected in three and *mcr*-3 in two of the five resistant isolates from pigs, and *mcr*-1 was found in the single resistant isolate from wastewater. In 2019 the single isolate from a breeder pig contained *mcr*-1. In 2020 *mcr*-1 positive *E. coli* isolates were found in all 5 piglets that had recent diarrhea symptoms and in wastewater samples (6/10).

### Antimicrobial Susceptibility Determination

**Figure 2:**
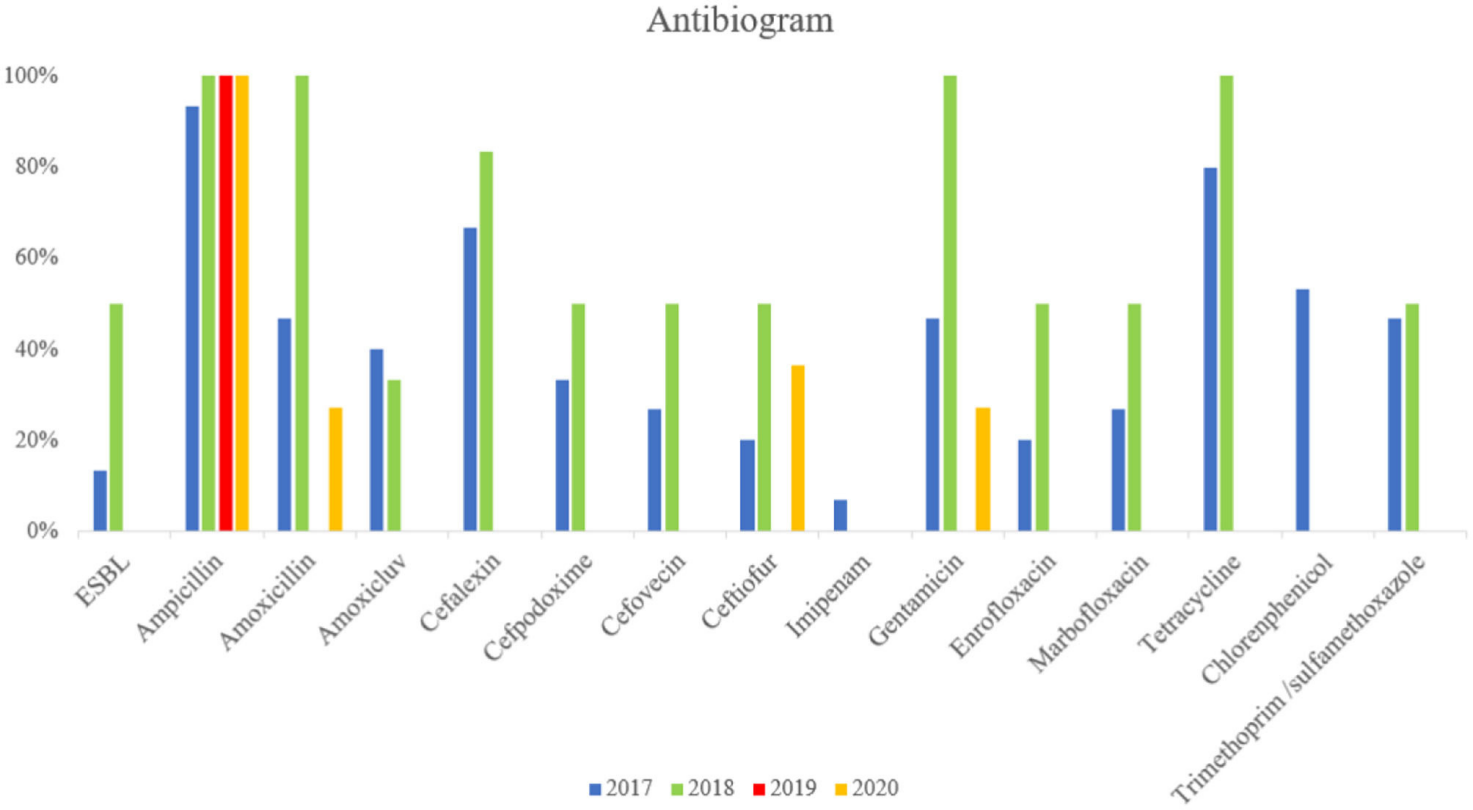
Comparison between resistance rates against 18 antimicrobials and extended-spectrum beta-lactamase (ESBL) in MCRPE isolates at four different sample collection times.

The antimicrobial resistance (AMR) profiles detected in the MCRPE isolates are shown in Figure 2. ESBL-producing *E. coli* were identified, and most MCRPE isolates from the first and second samplings were found to demonstrate extreme pan-drug resistance. Interestingly, besides colistin, the isolate from the positive pig sample in 2019 was phenotypically resistant only to ampicillin. On the other hand, the MCRPE isolates from the last sample collection in 2020 were resistant to aminoglycosides, ampicillin, and ceftiofur, and those antibiotics were used for individual treatments on the farm. The antibiogram results comparing isolates between the 4 sampling years are presented in Supplementary Table 1. High rates of resistance to ampicillin, gentamicin, and tetracycline were detected in almost all MCRPE isolates at each sampling time.

Various plasmid replicon types were detected among the MCRPE isolates (Table 3). All the *mcr* positive isolates from different sources contained more than one replicon type. The incompatibility group IncFIB and IncI type plasmids were found most commonly. Although a variety of plasmid types were detected in pigs in 2017 and 2018, there was a decrease in varieties of plasmid types in later sample collection years. For the conjugation assay, the donor *E. coli* transferred *mcr*-1 and *mcr*-3 genes (as confirmed by PCR) to recipient J53 strains with a frequency of 1.7–2 × 10^−4^. Phenotypic colistin resistance of transconjugants identified by broth microdilution showed MIC values of >4 μg/ml (Supplementary Table 2). The IncI, IncX and IncF plasmid types were predominantly detected in *mcr*-1 transconjugants, while the IncHI2 and IncF plasmid types were detected on *mcr*-3 transconjugants (data not showed).

**Table 3:** Plasmid replicon types detected in 33 colistin-resistant *E. coli* among the three categories of samples at each sample collection time.

**Trait**	**Pigs**				**Workers**	**Wastewater**		
	**2017**	**2018**	**2019**	**2020**	**2017**	**2017**	**2018**	**2020**
	***n =* 9**	***n =* 5**	***n =* 1**	***n =* 5**	***n =* 4**	***n =* 2**	***n =* 1**	***n =* 6**
I1-Ir	+	+	–	+	–	–	–	+
HI1	+	+	–	–	–	–	–	–
HI2	+	–	–	+	–	–	–	–
N	+	+	–	–	–	–	–	–
X	+	–	–	–	+	–	+	–
FIB	+	+	+	+	+	+	–	+
FIA	+	+	+	–	–	–	–	–
FIC	–	+	+	–	+	–	+	–
P	+	+	–	–	–	–	–	–
Y	+	+	–	+	+	+	–	+
A/C	+	–	–	+	+	–	–	–
I	–	+	+	+	–	+	+	–

*+, detected; –, not detected*.

### Virulence Gene Detection

Virulence gene detection was performed on all the 33 *mcr* positive *E. coli* isolates. Most of the isolates from pigs contained genes associated with ETEC strains (enterotoxin genes), with *StaP* and *Stb* being the most frequent pathotype found in 2017 (Table 2). One strain from a pig in 2017 showed a hybrid ETEC–EHEC genotype. Two of the four colistin-resistant *E. coli* recovered from farm workers in 2017 contained a combination of *StaP* and *Stb* genes. In contrast, the wastewater samples and the piglets' samples obtained after 2017 only contained the *Stb* enterotoxin gene.

### Molecular Genotypic Characterizations

**Figure 3:**
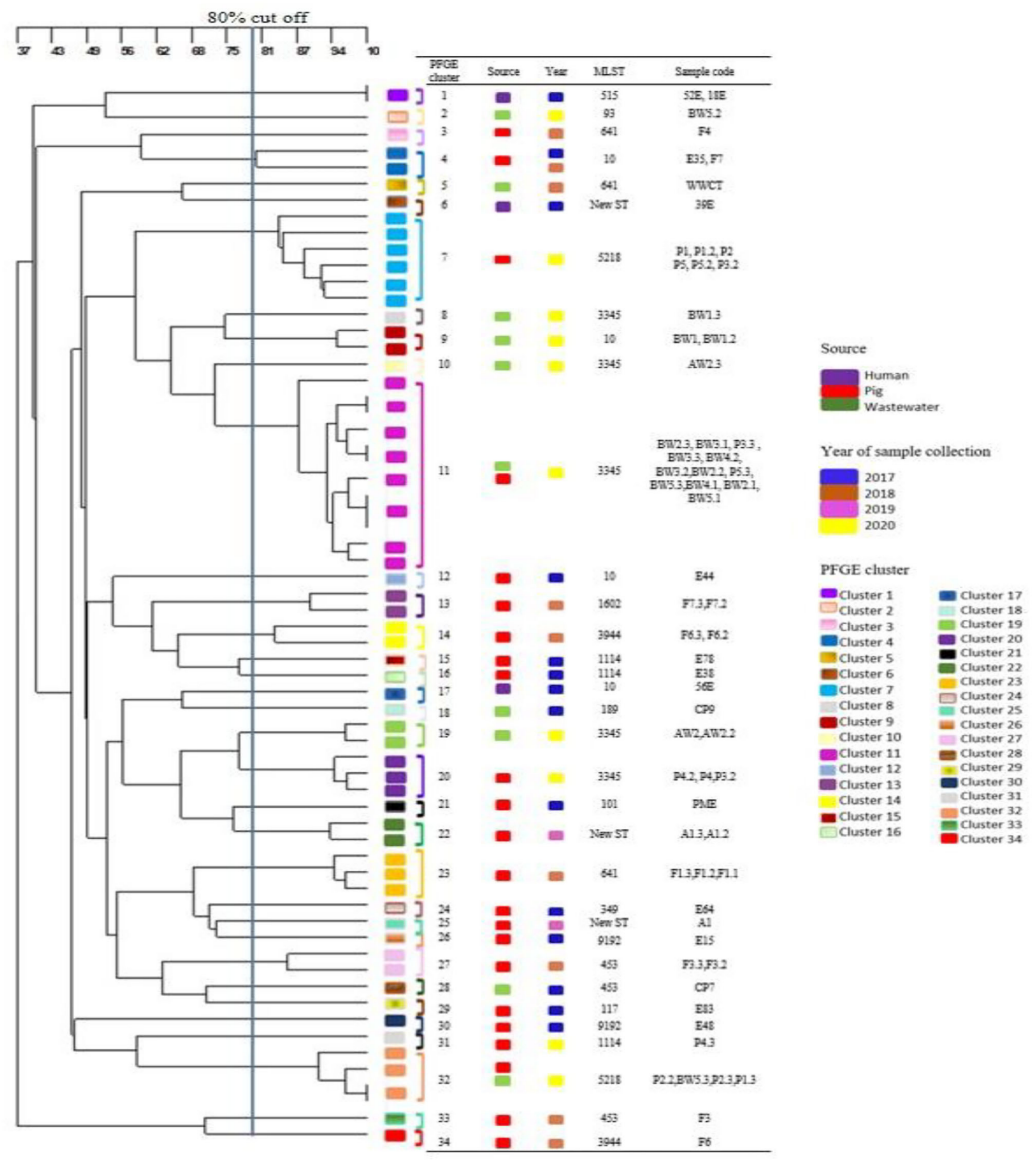
Dendrogram generated from pulsed field gel electrophoresis analysis demonstrating the genetic relatedness among 65 *mcr* positive *Escherichia coli* strains (one to three isolates per positive sample) that were obtained from different sources at each sampling time.

Thirty-four diverse PFGE patterns were obtained for the 65 MCRPE isolates from different sources (Figure 3). No dominant pulsotypes were responsible for *mcr* gene clonal carriage. Moreover, most of the strains from each sample collection time were dispersed on different branches of the dendrogram and were not closely related genetically. The pulsotypes of the MCRPE from humans were not clonally related to any of those from pigs or wastewater. Strains with high similarity (>80%) occurred rarely and were found mainly in the same set of pig or human samples from the same sampling year. Only the MCRPE strains from piglets and wastewater samples in 2020 showed high clonal relatedness, suggesting that MCRPE strains from the piglets with diarrhea had contaminated the wastewater.

MLST gave similar results to PFGE, with most isolates belonging to different STs (Supplementary Table 3). Isolates of the common *E. coli* clonal complex ST10 were detected in 2 pigs and one human sample on the first sampling, and in one wastewater sample on the last sample collection. Isolates belonging to ST 641 were detected in 2 pigs and one wastewater sample in 2018. In 2020, MCRPE isolates belonging to ST3345 (*n* = 3 in wastewater, *n* = 2 in pigs) and ST 5218 (*n* = 2 in pigs) were commonly detected.

## Discussion

The geographical distribution and characterization of colistin-resistant *E. coli* on large-scale pig farms across Thailand has been reported previously (19). The current longitudinal study investigated the persistence and diversity of *mcr* positive *E. coli* on a selected commercial Thai pig farm following withdrawal of prophylactic colistin usage. According to the farm history, batches of piglets previously were consistently prescribed colistin up until the time that it was withdrawn from use at the start of 2017. In searching for potential changes in resistance to colistin after its withdrawal, this study focused on examining colistin resistance in *E. coli* from young sows and their suckling pigs, as well as from wastewater. Prior to 2017 colistin was mainly used for controlling *E. coli* in suckling pigs in their first 3 weeks of life, so it seemed logical to target this bacterial species and this age group when looking for ongoing resistance. In addition, sows were examined since piglets become colonized by oral exposure from the fecal microbiota of their mothers. The sows were exposed to colistin prior to 2017 and might be persistently colonized and hence transfer resistant bacteria to their piglets. The piglets themselves were destined for slaughter by around 5–6 months of age, and so by definition could not be involved in direct transmission in following years. Accordingly, more sows than piglets were sampled to determine whether they still represented a potential long-term reservoir of MCRPE infection. Wastewater also was sampled, as wastewater tanks on pig farms serve as hotspots for accumulating resistant bacteria since they are composed of pooled fecal discharge from large numbers of pigs housed in the same area. Inclusion of this material in the study increased the likelihood of detecting MCRPE. Although relatively small numbers of samples were examined at each sampling time, they were sufficient to confirm the presence of MCRPE throughout the study.

Even following colistin withdrawal for 21 months, MCRPE that were carrying *mcr* genes were still quite commonly found in pigs, indicating that this period is insufficient to have a significant effect on reducing the presence of colistin resistant bacteria after the drug's withdrawal. The presence of *mcr* positive *E. coli* in the feces of farm workers in mid-2017 is a matter of considerable concern. Bacteria from animals can be transmitted to humans either directly or through food or the environment, and then may transfer resistance genes to pathogenic bacteria that infect humans (43). Farm and food chain workers are likely to be exposed to resistant bacteria throughout the pig production cycle (44). Moreover, *Stb* and *StaP* virulence genes were found in MCRPE isolates from pigs and in two of the workers. These enterotoxin genes are linked to neonatal or postweaning diarrhea in pigs, but bacteria carrying the genes also can be shed in feces from healthy animals (45). The *Stb* enterotoxin is commonly found in *E. coli* strains from pigs but is rarely found in humans and is not associated with diarrhea in humans (46, 47). Therefore, these findings suggested that subclinical ETEC carriers can be found at various stages in the pig production cycle and may represent a source of transmission to humans. Even though isolates of identical genetic types were not found in humans and pigs, our results highlight possible transmission of *mcr* genes from bacteria infecting livestock to isolates that are present in humans and in the environment. The failure to recover MCRPE from human samples after 2017 may be associated with reduced exposure to colistin and/or to MRCPE from pigs and the environment. One possibility is that following identification of MCRPE in the workers in 2017 these individuals took greater care of their hygiene to reduce their exposure to MCRPE of pig origin.

Most pigs were still colonized with colistin-resistant *E. coli* when sampled 21 months after colistin withdrawal; however, by the third year there was a sharp decline in carriage by pigs and neither workers nor wastewater samples were positive for MCRPE. Therefore, the national ban on prophylactic use of colistin was highly likely to have been beneficial for controlling the emergence and dissemination of *mcr*-1 on this and on other pig farms. Nevertheless, some pigs and wastewater still contained MRCPE when samples 3.5 years after the withdrawal of colistin. Pigs reared for meat production are only kept for around 5–6 months before slaughter, although breeder pigs are retained for up to 3–4 years. Presumably transmission cycles of MCRPE between batches of pigs that are selected for meat production or breeding, and/or exposure to contaminated environments allowed them to remain for at least 3.5 years. A more extended study is required to determine for how long this carriage my persist. The long duration of persistence that was identified contrasted with a previous report from Britain, where *mcr*-1 was undetectable in isolates from pigs after the cessation of colistin use for approximately 20 months (7). The reason for the re-occurrence and increase in numbers of pigs shedding MCRPE and in isolates recovered from wastewater in the last year of the current study is unclear. These colonized pigs had shown diarrhea symptoms prior to sampling and had been given therapeutic antimicrobial treatments, unlike the situation in previous batches sampled in earlier years. A possible explanation for the re-occurrence without selective pressure applied from colistin exposure may be the existence of cross-resistance between colistin and other therapeutic antibiotics used in the piglets. A similar phenomenon was reported in previous studies where colistin resistance was found when other antimicrobials such as quinolones or cephalosporins were used in livestock farms (48, 49). However, more complete genomic characterization of the MCRPE isolates involved is required to investigate possible reason for this correlation. Nevertheless, these results are of concern because short-term β-lactam (ceftiofur) or gentamicin use in animals may select for *mcr*-1 in *E. coli* and maintain persistence on farms.

From the antibiotic susceptibility testing, some of the *mcr* positive *E. coli* isolates were found to be ESBL producers and showed extreme pan-drug resistance. A larger number of *E. coli* isolates with ESBL were observed in the samples from 2018 compared to the first sampling time. In Thailand, the application of antimicrobials in pig farms varies according to the management system and geographical area. In the central area of Thailand, the antimicrobials that are mainly used are colistin, cephalosporins, tiamulin, amoxicillin, tilmicosin, aminoglycosides (gentamycin), and oxytetracycline (50). The use of other antimicrobials during the production cycle of pigs could co-select for colistin resistance (51, 52). Resistances to other potential agents like heavy metals or biocides that may be linked with antibiotics resistance genes also are a matter for concern.

In the conjugation experiment, MCRPE recovered from pigs without selective pressures from colistin use showed a high transfer frequency. Moreover, various replicon types were found in the colistin-resistant *E. coli* isolates. According to previous reports, *mcr-*1 and *mcr-*3 genes have been found on IncI, IncHI2, and IncX4 plasmids (53). Likewise, *mcr*-1 was predominantly harbored on the IncX4 plasmid in isolates from healthy human beings in China (54). Different AMR genes can be located on the same plasmid or on different plasmids within the same bacterial host, and these represent multidrug resistant clones. Plasmids encoding the *mcr* genes, which co-exist with other antimicrobial resistance genes, are a problem for public health. To date, the majority of *mcr* genes have been identified in various plasmid types and are able to locate and/or transfer with other resistance genes by conjugation (55).

A large number of PFGE pulsotypes were observed among the *mcr* positive isolates. Therefore, no epidemic strains were dominant on the farm over time, and the *mcr* genes found in *E. coli* isolates were mainly plasmid-borne. A high diversity of MCRPE isolates from different hosts also was observed in a study from China (56). Similarly, in a Dutch study where ESBL positive *E. coli* from animals and humans were examined, ESBL transmission did not involve strain transfer but rather plasmid transfer by identical plasmids of the IncI and IncK types (57). Nevertheless, in our study some clonal relatedness was found in MCRPE from piglets and wastewater samples at the last sampling. In this case the resistant bacteria from pigs were likely to be the primary source of *mcr* genes contaminating wastewater. Thus, despite moderate persistence of *mcr* genes in pigs and low-level environmental dissemination in tested wastewater, the distribution of diverse strains with virulence potential from different niches across years is worrisome. Genes from these *mcr*-1 and *mcr*-3 positive isolates might be transferred to other sources and/or other pathogens.

In this study, *E. coli* carrying *mcr* genes were recovered, but with a gradual decline over 3.5 years following cessation of colistin use. Hence, banning colistin for prophylaxis use was effective for reducing the emergence and dissemination of *mcr*-1 in pigs and the pig farm environment. However, even in the absence of selective pressure exerted by colistin use, the application of other antimicrobials during the production cycle might co-select indirectly for the *mcr* genes and favor their spread. This study provides an initial insight into the reduction in dissemination of colistin resistant *E. coli* from pigs and the farm environment. Further long-term genomic investigations are necessary to improve understanding and control of MCRPE and colistin-resistance in the pig industry.

## Data Availability Statement

The original contributions presented in the study are included in the article/Supplementary Material, further inquiries can be directed to the corresponding author/s.

## Ethics Statement

Ethical review and approval was not required for the animal study because All fecal samples were submitted from veterinarians in pig industrial field to the veterinary diagnostic laboratory as the annual surveillance. However, the biohazard execution control was approved by the Institutional Biosafety Committee of the Faculty of Veterinary Science, Chulalongkorn University (IBC 2031011).

## Author Contributions

NK contributed to conception and design, critical revision, analysis and interpretation of data, and drafting of manuscript. KL contributed to conception and design, analysis, and interpretation of data. WN performed analysis and interpretation of data. TP organized acquisition of data. DH contributed to critical revision, analysis and interpretation of data, and drafting of manuscript. NP organized study conception and design and contributed to critical revision, analysis, and interpretation of data. All authors contributed to manuscript revision, read, and approved the submitted version.

## Funding

This study was supported financially by Royal Golden Jubilee Ph.D. (RGJPHD) program, from the Agricultural Research Development Agency (ARDA) [CRP6205031110], the CHE-TRF Senior Research Fund (RTA6280013), Thailand Science Research and Innovation, and Pathogen Bank, Faculty of Veterinary Science, Chulalongkorn University, Bangkok, Thailand.

## Conflict of Interest

The authors declare that the research was conducted in the absence of any commercial or financial relationships that could be construed as a potential conflict of interest.

## Publisher's Note

All claims expressed in this article are solely those of the authors and do not necessarily represent those of their affiliated organizations, or those of the publisher, the editors and the reviewers. Any product that may be evaluated in this article, or claim that may be made by its manufacturer, is not guaranteed or endorsed by the publisher.
