# Supplementary material for: Longitudinal Monitoring Reveals Persistence of Colistin-Resistant Escherichia coli on a Pig Farm Following Cessation of Colistin Use
Source: Front Vet Sci. 2022 Mar 14;9:845746. doi: 10.3389/fvets.2022.845746 (PMC8964308; doi:10.3389/fvets.2022.845746)
Supplement: Supplementary file 1 [file Table_1.docx]

Supplementary table 1. Antibiogram patterns detected from the 33 MCRPE isolates from samples collected in four different years

| Year | | | Year | | | Year | |  | Year | | |
| --- | --- | --- | --- | --- | --- | --- | --- | --- | --- | --- | --- |
| **2017** | | | **2018** | | | **2019** | |  | **2020** | | |
|  | Number of ABOs resistant | Number of isolates |  | Number of ABOs resistant | Number of isolates |  | Number of ABOs resistant | Number of isolates |  | Number of ABOs resistant | Number of isolates |
| CEX-GEN-CST | 3 | 1 | AMX-AMP-PIP-CEX-GEN-TET-CST* | 7 | 2 | AMP-CST | 2 | 1 | AMP-TET-CST | 3 | 7 |
| AMX-AMP-TET-CST | 4 | 2 | AMX-AMP-AMC-PIP-CEX-GEN-ENR-MBR-TET-CST | 10 | 1 |  |  |  | AMX-AMP-XNL-TET-CST | 5 | 2 |
| AMX-AMP-GEN-TET-CST | 5 | 1 | AMX-AMP-PIP-CEX-CPD-INN-CEF-GEN-TET-SXT-CST* | 11 | 1 |  |  |  | AMX-AMP-XNL-GEN-TET-CST | 6 | 2 |
| AMX-AMP-PIP-TET-C-CST | 6 | 1 | AMX-AMP-PIP-CEX-CPD-INN-CEF-GEN-ENR-MBR-TET-SXT-CST* | 13 | 1 |  |  |  |  |  |  |
| AMX-AMP-PIP-TET-SXT-C-CST | 7 | 1 | AMX-AMP-AMC-PIP-CEX-CPD-INN-CEF-GEN-ENR-MBR-TET-SXT-CST | 14 | 1 |  |  |  |  |  |  |
| AMP-AMC-CEX-CEF-C-SXT-CST | 7 | 1 |  |  |  |  |  |  |  |  |  |
| AMP-CEX-CEF-GEN-TET-CST | 7 | 1 |  |  |  |  |  |  |  |  |  |
| AMX-AMP-AMC-PIP-CEX-CPD-TET-CST | 8 | 1 |  |  |  |  |  |  |  |  |  |
| AMP-AMC-CEX-CEF-CPD-INN-TET-C-SXT-CST | 10 | 1 |  |  |  |  |  |  |  |  |  |
| AMP-CEX-CEF-GEN-ENR-MBR-TET-C-SXT-CST | 10 | 1 |  |  |  |  |  |  |  |  |  |
| AMP-AMC-CEX-CEF-GEN-ENR-MBR-TET-C-SXT-CST* | 11 | 1 |  |  |  |  |  |  |  |  |  |
| AMP-CEX-CEF-CPD-INN-GEN-MBR-TET-SXT | 11 | 1 |  |  |  |  |  |  |  |  |  |
| AMX-AMP-AMC-PIP-CEX-CPD-INN-CEF-IMP-C-SXT-CST | 12 | 1 |  |  |  |  |  |  |  |  |  |
| AMP-AMC-CEX-CEF-CPD-INN-GEN-ENR-MBR-TET-C-CST* | 13 | 1 |  |  |  |  |  |  |  |  |  |

AMC, amoxicillin–clavulanic acid; AMP, ampicillin; AMX, amoxicillin; C, chloramphenicol; CEX, cephalexin; CPD, cefpodoxime; ENR, enrofloxacin; GEN, gentamicin; MBR, marbofloxacin; PIP, piperacillin; SXT, trimethoprim/sulfamethoxazole; INN, cefovecin; AK, amikacin; IMP, imipenem; TET, tetracycline; XNL, ceftiofur; TOB, tobramycin; NIT, nitrofurantoin, CST, colistin; * =ESBL

Supplementary Table 2. The minimum inhibitory concentration of colistin and plasmid replicon profiles of transconjugants

| Donor Strains | | Total number of isolates | Recipient *E. coli* J53 ( Transconjugants) | | |
| --- | --- | --- | --- | --- | --- |
|  |  |  | Colistin MIC  (µg/ml) | *mcr* genes | Plasmid (Inc) types determined (n) |
| Farrowing Sows | | 15 | >4 | *+* | IncFIB (6), IncFIC (3), IncFIA (1), IncI (4), IncHI2 (2) |
| Piglets | | 5 | >4 | *+* | IncI (3), IncHI2 (1), IncFIB (1) |
| Human | | 4 | >4 | *+* | IncX (3), IncFIB (1),  IncI (1) |
| Wastewater | | 9 | >4 | *+* | IncFIB (4), IncI (1),  IncX (1), IncY (3) |
|  |  |  |  |  |  |

Supplementary table 3. MLST sequence types and source and number of the MCRPE isolates that were representatives of the 34 PFGE patterns

| Year | Sequence Type | Source | Number of isolates tested |
| --- | --- | --- | --- |
| 2017 | **10** | Pig | 2 |
|  | **10** | Human | 1 |
|  | **101** | Pig | 1 |
|  | **349** | Pig | 1 |
|  | **9192** | Pig | 2 |
|  | **117** | Pig | 1 |
|  | **1114** | Pig | 2 |
|  | **453** | Wastewater | 1 |
|  | **189** | Wastewater | 1 |
|  | **515** | Human | 1 |
|  | **New ST** | Human | 1 |
| 2018 | **641** | Pig | 2 |
|  | **641** | Wastewater | 1 |
|  | **3944** | Pig | 2 |
|  | **1602** | Pig | 1 |
|  | **453** | Pig | 2 |
| 2019 | **New ST** | Pig | 2 |
| 2020 | **3345** | Pig | 2 |
|  | **3345** | Wastewater | 3 |
|  | **5218** | Pig | 2 |
|  | **1114** | Pig | 1 |
|  | **10** | Wastewater | 1 |
|  | **93** | Wastewater | 1 |
